# Utilizing the Centiloid scale in cross-sectional and longitudinal PiB PET studies

**DOI:** 10.1016/j.nicl.2018.04.022

**Published:** 2018-04-25

**Authors:** Yi Su, Shaney Flores, Russ C. Hornbeck, Benjamin Speidel, Andrei G. Vlassenko, Brian A. Gordon, Robert A. Koeppe, William E. Klunk, Chengjie Xiong, John C. Morris, Tammie L.S. Benzinger

**Affiliations:** a Department of Radiology, Washington University School of Medicine, Saint Louis, MO 63110, USA; b Department of Neurology, Washington University School of Medicine, Saint Louis, MO 63110, USA; c Knight Alzheimer Disease Research Center, Washington University School of Medicine, Saint Louis, MO 63110, USA; d Division of Biostatistics, Washington University School of Medicine, Saint Louis, MO 63110, USA; e Department of Radiology & Biomedical Imaging, University of California, San Francisco, CA, USA; f Department of Radiology, University of Michigan, Ann Arbor, MI, USA; g Department of Neurology, University of Pittsburgh, Pittsburgh, PA, USA

**Keywords:** PET, PiB, Amyloid imaging, Centiloid

## Abstract

Amyloid imaging is a valuable tool for research and diagnosis in dementing disorders. Successful use of this tool is limited by the lack of a common standard in the quantification of amyloid imaging data. The Centiloid approach was recently proposed to address this problem and in this work, we report our implementation of this approach and evaluate the impact of differences in underlying image analysis methodologies using both cross-sectional and longitudinal datasets. The Centiloid approach successfully converts quantitative amyloid burden measurements into a common Centiloid scale (CL) and comparable dynamic range. As expected, the Centiloid values derived from different analytical approaches inherit some of the inherent benefits and drawbacks of the underlying approaches, and these differences result in statistically significant (*p* < 0.05) differences in the variability and group mean values. Because of these differences, even after expression in CL, the 95% specificity amyloid positivity thresholds derived from different analytic approaches varied from 5.7 CL to 11.9 CL, and the reliable worsening threshold varied from −2.0 CL to 11.0 CL. Although this difference is in part due to the dependency of the threshold determination methodology on the statistical characteristics of the measurements. When amyloid measurements obtained from different centers are combined for analysis, one should not expect Centiloid conversion to eliminate all the differences in amyloid burden measurements due to variabilities in underlying acquisition protocols and analysis techniques.

## Highlights

• The Centiloid approach brings amyloid burden measurements into a common scale.
• The Centiloid value inherits the characteristics of the underlying method.
• The Centiloid value derived from different analysis techniques remains different.
• The amyloid positivity thresholds in Centiloid are sensitive to implementation.

## 1 Introduction

Alzheimer's disease (AD) is the most common form of dementia (Holtzman et al., 2011) and the prevalence of AD is expected to increase dramatically within the next 50 years (Alzheimer's, 2014). Currently, there are no proven disease-modifying treatments (Aisen, 2009; Aisen et al., 2011; Doody et al., 2013; Huang and Mucke, 2012); further research and development are in urgent need to prevent and/or treat this disease. It is well established that AD pathologies including amyloid plaques and neurofibrillary tangles begin to accumulate well before clinical symptoms appear (Bateman et al., 2012; Benzinger et al., 2013; Holtzman et al., 2011; Jack et al., 2010; Jansen et al., 2015; Morris and Price, 2001). Therefore, there is an increasing consensus that early intervention is necessary to effectively treat AD or slow down its progression (Aisen, 2009; Aisen et al., 2011). To enable the design of therapeutic trials, especially in asymptomatic individuals, validated surrogate biomarkers for AD pathology are necessary (Aisen, 2009; Aisen et al., 2011). As the primary pathological process in AD, accurate assessment of amyloid deposition in the brain may serve as an effective biomarker for the investigation of AD and marker in treatment trials.

To achieve this goal, positron emission tomography (PET) imaging tracers such as [^11^C]Pittsburgh Compound-B (PiB) (Klunk et al., 2004), [^18^F]florbetapir (Wong et al., 2010), [^18^F]florbetaben (Rowe et al., 2008) and [^18^F]flutemetamol (Vandenberghe et al., 2010), were developed to enable in vivo measurement of fibrillar beta-amyloid (Aβ) deposition. However, differences in these imaging tracers can lead to different estimations of the amyloid burden in the brain (Klunk et al., 2015; Landau et al., 2013). In addition to tracer differences, there is also substantial variability in the methods different groups use to quantify amyloid burden (Su et al., 2016), leading to difficulties in comparing and interpreting numeric results reported from different groups (Klunk et al., 2015).

To address these issues and facilitate standardization of PET based amyloid burden measurements, the Centiloid Working Group outlined the procedures for establishing the Centiloid scale and converting group specific amyloid burden measurements into the standard scale (Klunk et al., 2015). This group also made the dataset used for defining the Centiloid scale publicly available at the Global Alzheimer's Association Information Network (GAAIN; http://www.gaain.org). In this approach, two anchor points were used to define the Centiloid scale: the mean amyloid burden of the young control (YC) group who are assumed to have no amyloid pathology in their brain (defined as 0 in the Centiloid scale) and the mean amyloid burden of the AD group in the GAAIN dataset (defined as 100 in the Centiloid scale). A standard image analysis procedure estimating the standard uptake value ratio (SUVr) of a global cortical target region (CTX) over whole cerebellum (WC) for PiB PET images acquired within the 50 to 70 min post-injection time window was described to calculate the standard Centiloid SUVr, which was then mapped to the Centiloid scale based on the two anchor points. The outcome measure of any other analysis techniques can then be converted to the Centiloid scale using a linear transformation based on the GAAIN dataset (or other datasets that meets the criteria for Centiloid calibration), i.e. level-2 calibration (Klunk et al., 2015). The initial Centiloid paper also described the requirements and steps to scale amyloid burden measurements obtained using other PET tracers to the Centiloid scale (Klunk et al., 2015). Since its inception, the research community has gradually adopted the Centiloid approach (Jack et al., 2017; Leuzy et al., 2016; Weiner et al., 2017), and calibrations of [^18^F]-NAV4694 (Rowe et al., 2016) and [^18^F]-florbetaben (Rowe et al., 2017) based amyloid burden measurements to the Centiloid scale have been published recently.

The goal of the Centiloid scale is to standardize PET based amyloid burden measurements to make comparisons of results from different research groups easier and facilitate the use of amyloid PET imaging as a diagnostic tool. However, it remains unknown how comparable Centiloid values derived from different analysis pipelines are, and what the impact of variability in the implementation of Centiloid analysis will be to cross-sectional and longitudinal studies. To answer these questions, we compared Centiloid values obtained from different analysis techniques using the GAAIN dataset and PiB PET imaging data from Knight Alzheimer Disease Research Center (ADRC) Memory and Aging Project. Specifically, 1) the GAAIN dataset was used to establish Centiloid conversion equations for 13 different methods to quantify global amyloid burden using PiB PET and then used to compare the variability of the measured amyloid burden within young controls who have no amyloid in their brain; 2) the impact of quantification methods to cross-sectional amyloid burden measurements after the Centiloid conversion was further evaluated in the Knight ADRC cohort; 3) longitudinal Knight ADRC data was used to examine the variability of amyloid burden measurements and assess the sensitivity to longitudinal changes in amyloid burden; and finally 4) we estimated amyloid positivity threshold in Centiloid scale and compared the thresholds obtained from different quantification methods.

## 2 Methods

### 2.1 Participants

The dataset used to define the Centiloid scale (downloaded from the GAAIN website: http://www.gaain.org) consists of 34 YCs (age ≤ 45 yrs) and 45 clinically diagnosed AD patients ages 50 to 89 who had a clinical dementia rating (CDR) (Morris, 1993) >0. A subset (GAAIN_SUB) of the GAAIN dataset (18 YCs and 18 ADs) had sufficient dynamic PiB PET data to allow all of our analysis techniques (e.g., binding potential calculation) to be performed. These numbers exceeded the number of participants recommended by the Centiloid working group for level-2 calibration (Klunk et al., 2015)) and were successfully processed with our local processing pipeline (Su et al., 2015; Su et al., 2013) and passed quality control.

The Knight ADRC cohort included 590 participants with at least a single PiB PET session, with a mean age of 67.7 ± 10.0 yrs., 37.6% APOE4 carriers, and 91 of them were CDR positive (>0). A subset of 243 participants had two longitudinal PiB PET data points with a mean baseline age of 65.8 ± 9.4 yrs., 32.5% of them were APOE4 carriers, and 16 of them were CDR positive. The mean follow-up interval was 3.2 ± 1.5 yrs.

#### 2.1.1 Ethics statement

All assessment and imaging procedures were approved by Washington University's Human Research Protection Office. Written informed consent was obtained from all individuals or their authorized representatives.

### 2.2 Imaging

The imaging protocols for the GAAIN dataset have been described previously (Klunk et al., 2015). The PiB PET from the GAAIN dataset includes PET images acquired within the 50–70 min post-injection window at a minimum. The GAAIN_SUB dataset had full dynamic multi-frame PET imaging data acquired between 0 and 70 min after injection of PiB. T1-weighted MRI was also available to provide anatomical information and facilitate PET quantification.

For the Knight ADRC cohort, dynamic PET imaging was conducted for 1 h with a Siemens/CTI EXACT HR+ scanner or a Biograph 40 PET/CT scanner (Siemens Medical Solutions, Erlangen, Germany) in three-dimensional mode after intravenous administration of approximately 12 mCi of PiB. Anatomic MRI was acquired with a T1-weighted magnetization-prepared rapid gradient echo (MPRAGE) sequence using a Siemens 1.5 T or 3 T scanner.

### 2.3 Image analysis

Standard Centiloid processing was performed on the GAAIN dataset as described in the initial Centiloid paper (Klunk et al., 2015). In summary, a summed PET image of the 50–70 min post-injection window was created from raw PET data. Both PET and MRI data for each subject were re-oriented to match the Montreal Neurological Institute (MNI)-152 T1-weighted template provided with the Statistical Parametric Mapping version 8 (SPM8) software (Ashburner, 2009). Subjects' MRIs were then coregistered to the MNI template and subsequently, the PET images were coregistered to the individual MRI. Spatial normalization was performed using the unified segmentation method (Ashburner and Friston, 2005) implemented in SPM8 to allow quantification in the MNI-152 atlas space. Standard Centiloid SUVr was calculated based on the CTX region and WC region described in (Klunk et al., 2015).

In addition to standard Centiloid processing, our local processing pipeline (PUP; https://github.com/ysu001/PUP) was also used to process the GAAIN_SUB dataset and Knight ADRC data. Details of PUP processing have been discussed previously (Su et al., 2015; Su et al., 2013). Standard FreeSurfer (v5.3; Martinos Center for Biomedical Imaging, Charlestown, Massachusetts, USA; https://surfer.nmr.mgh.harvard.edu/fswiki) based PUP processing (Su et al., 2015; Su et al., 2013) includes scanner resolution harmonization filter (Joshi et al., 2009), inter-frame motion correction, PET-MR registration, regional time-activity curves extraction, regional spread function (RSF) based partial volume correction (PVC) (Rousset et al., 2008; Su et al., 2015), binding potential estimated using Logan graphical analysis (BP_ND_) (Logan et al., 1996), and SUVr analysis. Cerebellar cortex was used as the default reference region for BP_ND_ and SUVr analysis with 30–60 min post-injection as the standard time window. As the global index of amyloid burden, a mean cortical binding potential (Mintun et al., 2006) or mean cortical SUVr was calculated based on a selected set of FreeSurfer-defined frontal, parietal, temporal and precuneus cortical regions (Su et al., 2013).

### 2.4 Quantification methods

In this study, a total of 13 different quantification methods were implemented to quantify amyloid burden (summarized in Table 1). In addition to the standard Centiloid processing described in the previous section, 12 additional methods were included in this study that varied in 1) quantitative metric, i.e. BP_ND_ vs. SUVr; 2) with or without RSF PVC; 3) reference regions, i.e. cerebellar cortex, brain stem, or whole cerebellum; 4) post-injection time window, i.e. 30–60 min vs. 40–70 min. The inclusion of various methods was motivated by several observations. Firstly, it has been suggested (van Berckel et al., 2013) that a more quantitative measurement such as BP_ND_ was more reliable in assessing amyloid burden than the commonly used SUVr measurements–although such a measurement requires longer scans and more complicated modeling. Secondly, whether PVC is beneficial to amyloid PET quantification remains a question of debate. For example, while we reported improved sensitivity to amyloid burden changes when RSF PVC was used (Su et al., 2015), another group did not observe the same benefit when using a similar PVC technique (Schwarz et al., 2016). Thirdly, it has been suggested that alternative reference regions may be more appropriate under various circumstances (Bateman et al., 2012; Benzinger et al., 2013; Edison et al., 2012) and may lead to better sensitivity to changes in amyloid burden (Chen et al., 2015; Schwarz et al., 2016; Su et al., 2016). Therefore, SUVr analysis was also performed using brainstem and whole cerebellum as the reference. Finally, due to differences in PET imaging protocols, different studies differ in the post-injection time window than what is available for analysis. For example, for the Knight ADRC cohort, PiB PET imaging stops at 60 min post-injection, while for the Dominantly Inherited Alzheimer's Network (DIAN) study (Morris et al., 2012), PiB PET data in the 40–70 min post-injection window is the standard. Therefore, analyses using both 30–60 min and 40–70 min post-injection window were performed for the GAAIN_SUB dataset.

**Table 1:** Amyloid burden measurements included in this study. The cerebellar cortex is used as the default reference region.

Amyloid burden measurements	In Centiloid scale	Notes
Centiloid SUVr	CL_WUSTL	WUSTL implementation of standard Centiloid processing using CTX as the target region and WC as the reference region
PiB_3060_BP	CL_3060_BP	Binding potential measurements derived from PUP processing using 30–60 min post-injection window without partial volume correction
PiB_3060_BP_RSF	CL_3060_BP_RSF	Binding potential measurements derived from PUP processing using 30–60 min post-injection window with partial volume correction
PiB_4070_BP	CL_4070_BP	Binding potential measurements derived from PUP processing using 40–70 min post-injection window without partial volume correction
PiB_4070_BP_RSF	CL_4070_BP_RSF	Binding potential measurements derived from PUP processing using 40–70 min post-injection window with partial volume correction
PiB_3060_SUVr	CL_3060_SUVr	SUVr measurements derived from PUP processing using 30–60 min post-injection window without partial volume correction
PiB_3060_SUVr_RSF	CL_3060_SUVr_RSF	SUVr measurements derived from PUP processing using 30–60 min post-injection window with partial volume correction
PiB_4070_SUVr	CL_4070_SUVr	SUVr measurements derived from PUP processing using 40–70 min post-injection window without partial volume correction
PiB_4070_SUVr_RSF	CL_4070_SUVr_RSF	SUVr measurements derived from PUP processing using 40–70 min post-injection window with partial volume correction
PiB_3060_SUVr_BS	CL_3060_SUVr_BS	SUVr measurements derived from PUP processing using 30–60 min post-injection window without partial volume correction using brainstem as the reference region
PiB_3060_SUVr_RSF_BS	CL_3060_SUVr_RSF_BS	SUVr measurements derived from PUP processing using 30–60 min post-injection window with partial volume correction using brainstem as the reference region
PiB_3060_SUVr_WC	CL_3060_SUVr_WC	SUVr measurements derived from PUP processing using 30–60 min post-injection window without partial volume correction using the whole cerebellum as the reference region
PiB_3060_SUVr_RSF_WC	CL_3060_SUVr_RSF_WC	SUVr measurements derived from PUP processing using 30–60 min post-injection window with partial volume correction using the whole cerebellum as the reference region

Based on the GAAIN_SUB dataset, Centiloid conversion equations were generated according to the level-2 analysis guidelines described in the initial Centiloid paper (Klunk et al., 2015) for each version of the global amyloid burden index generated by our local analysis pipeline. Estimated amyloid burdens were then converted into Centiloid units (CL) for each variation of the global amyloid index for both GAAIN_SUB dataset and ADRC data using those equations. Knight ADRC data was not processed with the standard Centiloid procedure because of the lack of PET data between 60 and 70 min post-injection.

### 2.5 Amyloid positivity thresholds

Three methods were used to define the thresholds for amyloid positivity in Centiloid scale. The first method simply transformed the empirical thresholds we have commonly used in the past, i.e. a mean cortical BP_ND_ of 0.18 (Su et al., 2013; Vlassenko et al., 2011) and a mean cortical SUVr_RSF of 1.42 (Sutphen et al., 2015; Vlassenko et al., 2016), which was an equivalent threshold as the mean cortical BP_ND_ = 0.18, into CL by applying the conversion equation determined in 2.3 & 2.4. The second method adopted the specificity thresholds (Jack et al., 2017) defined as the 95th percentile of the amyloid burden measurements among the subset of YC participants in the GAAIN_SUB dataset. The last method used a modified “reliable worsening” (RW) approach (Jack et al., 2017), in which the threshold was defined as the amyloid burden at which the smoothed rate-of-change to baseline amyloid burden curve went above zero after the curve passes the minimum. The rate to baseline amyloid burden curve was generated based on the ADRC longitudinal dataset. A locally weighted regression method (LOESS) (Cleveland, 1979) was used to smooth the raw data, and bootstrapping was performed to obtain the confidence interval of the LOESS curves. This modification was necessary because using the original RW approach (Jack et al., 2017) for some analysis techniques we examined, the estimated threshold corresponds to a negative rate of amyloid accumulation, which was against the definition of worsening amyloid pathology (see Fig. 4 for an example).

### 2.6 Statistical analysis

The mean and standard deviation of the estimated amyloid burden in CL for the YC participants (*N* = 18) in the GAAIN_SUB dataset were calculated and used to assess the inter-individual variability of PiB-PET measure in healthy participants as an indicator of the reliability of the amyloid burden measurements. A threshold (CL = 10.7) was determined based on the 99th percentile of CL value obtained using FreeSurfer-defined whole cerebellum as the reference region with no partial volume correction, using 30–60 min window and SUVr analysis (CL_3060_SUVr_WC). This threshold was used to group Knight ADRC participants into amyloid negative and amyloid positive groups for additional analysis, and it was intentionally chosen to be different from the thresholds determined in 2.4 to avoid circularity. It was more conservative than the conventional thresholds we used previously, i.e. mean cortical BP_ND_ = 0.18 & mean cortical SUVr_RSF = 1.42, to minimize the possibility of including participants who have very low levels of amyloid. According to this threshold, the Knight ADRC longitudinal dataset has 182 stable amyloid negative participants who remained amyloid negative at both baseline and follow-up, and 61 participants (accumulators) were either amyloid positive at baseline or converted to amyloid positive at follow-up. F-tests were used to determine whether there were differences in the variability of amyloid burden measurements obtained from two different techniques. Paired *t*-tests were used to determine whether there were significant differences in the mean CL values when different analysis techniques were used. The longitudinal data for stable amyloid negative participants in the Knight ADRC cohort was assumed to have minimal changes in amyloid burden in the follow up to assess the intra-individual variability of the amyloid burden measurements. For amyloid accumulators in the longitudinal Knight ADRC cohort (*N* = 61), paired t-tests were used to assess the significance of longitudinal change, an effect size measure was also calculated as the ratio of annualized rate-of-change over intra-individual variability estimated based on the amyloid negative participants to assess the statistical power of CL derived from a particular analysis technique. To assess the impact of the difference in statistical power, similar to our previous study (Su et al., 2016), we also estimated the number of participants per arm needed to detect a 50% reduction in amyloid accumulation rate due to treatment with 80% power and a two-tailed type-I error of *p* = 0.05 in a hypothetical 12-month placebo-controlled randomized clinical trial. A total of 84 hypothesis tests were performed in our analysis, to account for multiple comparisons, an overall significance level was defined as *p* < 0.0005 (i.e. after Bonferroni correction of 100 comparisons), while a tentative significance level was set at *p* < 0.05 (i.e. without correction for multiple comparisons).

## 3 Results

### 3.1 Centiloid analysis in the GAAIN dataset

**Fig. 1:**
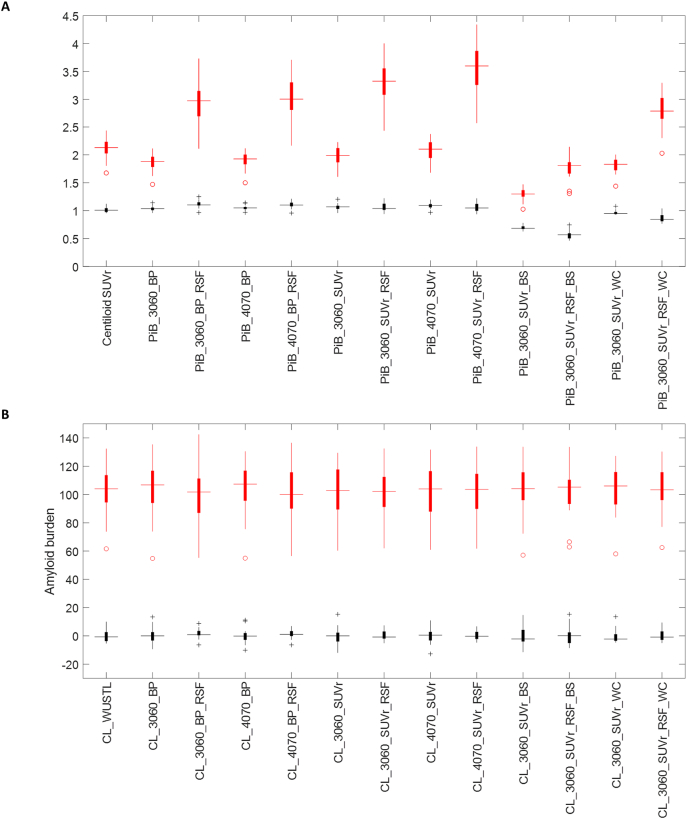
Box plot of amyloid burden measurements for the GAAIN dataset before (A) and after (B) converting to the Centiloid scale. YC data are shown in black and AD data are shown in red. The same 13 versions of the amyloid burden measurements summarized in Table 1 were included in this figure. Binding potential data are presented as BP + 1 in (A) to allow better comparison with SUVr measurements.

Our implementation of the standard Centiloid analysis generated results in strong agreement with the published Centiloid measurements for the GAAIN dataset (r^2^ = 0.9996) (Supp. Fig. 1). The conversion equations for the 12 different implementations of PiB-PET analysis techniques and details of the analysis results are given in the supplementary material. Without the conversion, the amyloid burden measurements obtained from different quantification methods varied in numerical values and dynamic ranges and could not be directly compared to each other (Fig.1A). The Centiloid conversion approach successfully converted these different measurements into approximately the same scale (Fig. 1B). After the Centiloid conversion, the variability across the YC group (V_YC_) was lower in general when RSF PVC was applied, although it did not reach statistical significance in some cases (Table 2). Other comparisons of V_YC_ for different analysis techniques, i.e. time window differences, SUVr vs BP_ND_, and choice of reference regions, in general did not reach significance (Table 2). Differences in analysis techniques (especially when different time windows were used for quantification) also had an impact on the mean CL values for the YC group and this was often amplified by RSF PVC (Table 2).

**Table 2:** Summary of Centiloid analysis on the GAAIN dataset.

	CL_WUSTL	CL_3060_ BP	CL_3060_ BP_RSF	CL_4070_ BP	CL_4070_ BP_RSF	CL_3060_ SUVr	CL_3060_ SUVr_RSF	CL_4070_ SUVr	CL_4070_ SUVr_RSF	CL_3060_ SUVr_BS	CL_3060_ SUVr_RSF _BS	CL_3060_ SUVr_WC	CL_3060_ SUVr_RSF _WC
YC mean	−0.1	0.0	1.6	0.0	1.2	−0.3	0.2	−0.2	0.3	−0.1	0.1	−0.4	0.1
YC SD	4.2	5.6	3.3	5.3	3.1	6.1	3.7	5.2	3.3	7.3	6.1	4.8	4.0
AD mean	102.9	102.8	101.2	102.8	101.6	103.1	102.6	103.0	102.5	102.9	102.7	103.2	102.7
R^2^		0.96	0.94	0.97	0.95	0.98	0.98	0.99	0.98	0.98	0.97	0.98	0.97
Specificity threshold (95%)													
	6.8	9.2	7.1	8.7	6.2	9.8	6.2	8.3	5.7	11.9	10.2	7.5	6.7
Level of significance													
Inter-individual variability													
RSFvsNonRSF			^⁎^		^⁎^		^⁎^		ns		ns		ns
BSvsCER										ns	^⁎^		
WCvsCER												ns	ns
4070vs3060				ns	ns			ns	ns				
SUVrvsBP						ns	ns	ns	ns				
Comparison of YC mean													
RSFvsNonRSF			ns		ns		ns		ns		ns		ns
BSvsCER										ns	ns		
WCvsCER												ns	ns
4070vs3060				^⁎⁎^	^⁎⁎^			^⁎⁎^	^⁎⁎^				
SUVrvsBP						ns	^⁎^	ns	^⁎^				

ns: nonsignificant; ^⁎^: *p* < 0.05; ^⁎⁎^: *p* < 0.0005, therefore remain significant after Bonferroni correction for multiple comparisons. The lower portion of the table reports the significance level for pair-wise comparison between different quantification techniques. For comparisons between RSF partial volume corrected methods with their counterparts without RSF PVC (RSFvsNonRSF), the significance level is reported under the RSF column; for comparisons between brainstem referencing methods with their counterparts with the default cerebellar gray referencing (BSvsCER), the significance level is reported under the BS column; for comparisons between whole cerebellum referencing methods with their counterparts with the default cerebellar gray matter referencing (WCvsCER), the significance level is reported under the WC column; For time window comparison (4070vs3060), the significance level is reported under the 4070 column; for comparison between SUVr and BP measurements (SUVrvsBP), the significance level is reported under the SUVr column. It should be noted that comparison for a particular technical variability was only performed between otherwise equivalent techniques.

### 3.2 Cross-sectional knight ADRC cohort analysis

**Fig. 2:**
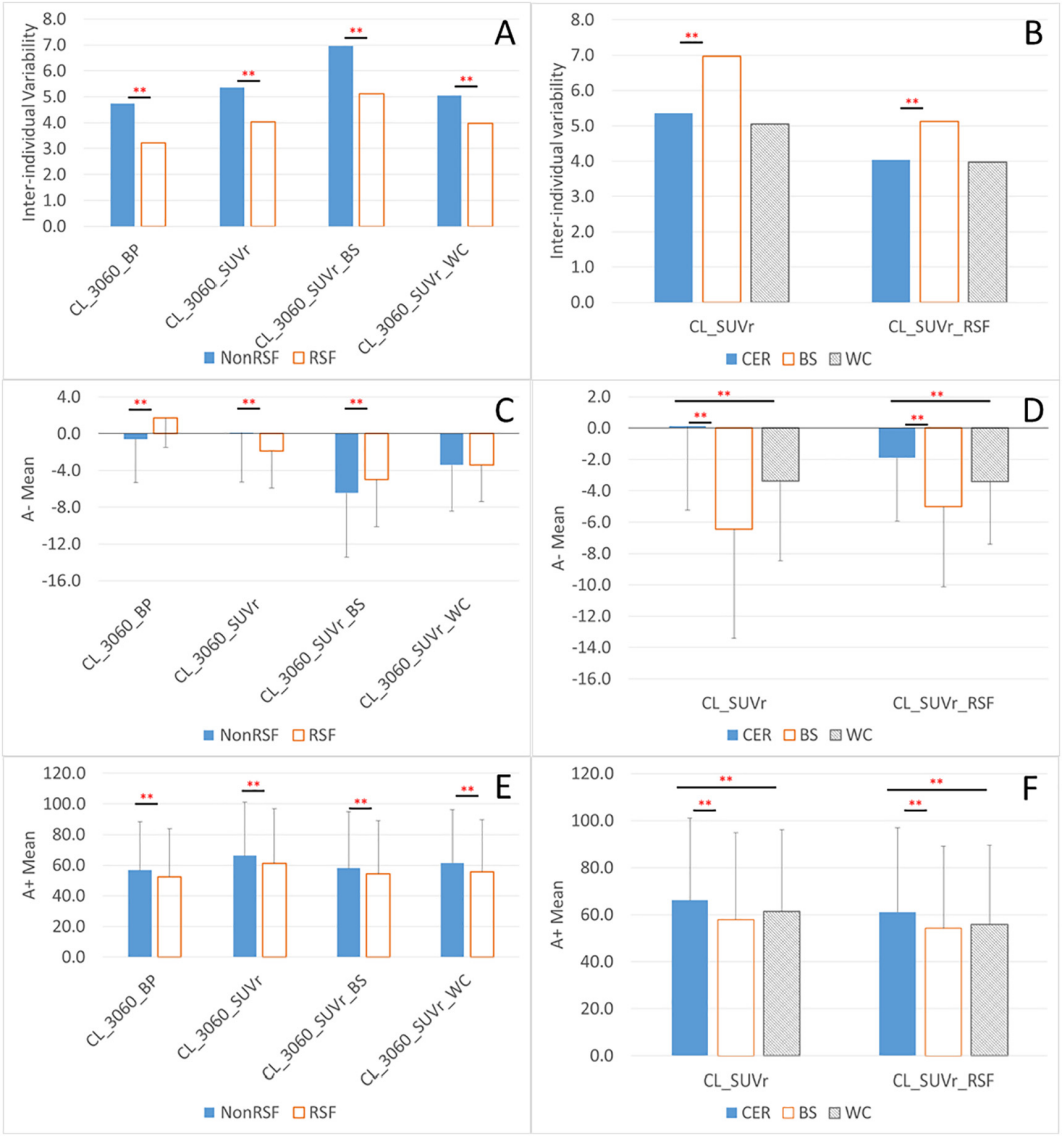
Comparison of inter-individual variability of measured amyloid burden (A, B), mean amyloid burden in people with minimal amyloid burden (C, D), and in people with substantial amyloid (E, F), for different quantification methods, i.e. with or without partial volume correction (A, C, E), and using different reference region (B, D, F). RSF: Regional spread function based partial volume correction; NonRSF: without partial volume correction; **significant difference (*p* < 0.0005) that survives Bonferroni correction for multiple comparisons. The analysis was based on the cross-sectional analysis of Knight ADRC data.

Results for the cross-sectional analysis of the Knight ADRC cohort are summarized in Table 3 and Fig. 2. In this larger cohort, it was demonstrated that differences in analytic techniques could have a significant impact (with a few exceptions) to both the variability in the Centiloid measurements, as well as group, mean values (Table 3, Fig. 2).

**Table 3:** Summary of Centiloid analysis on the cross-sectional ADRC cohort.

	CL_3060_BP	CL_3060_ BP_RSF	CL_3060_ SUVr	CL_3060_ SUVr_RSF	CL_3060_ SUVr_BS	CL_3060_ SUVr_RSF _BS	CL_3060_ SUVr_WC	CL_3060_ SUVr_RSF _WC
A− Mean	−0.6	1.7	0.1	−1.9	−6.4	−5.0	−3.4	−3.4
A− SD	4.7	3.2	5.4	4.0	7.0	5.1	5.1	4.0
A+ Mean	56.8	52.5	66.2	61.2	58.0	54.2	61.5	55.8
A+ SD	31.6	31.2	34.9	35.9	36.8	35.0	34.7	33.8
RW threshold								
	10.0	6.0	11.0	2.0	−2.0	−2.0	6.0	0.0
Specificity threshold (95%) (duplicated from Table 2)								
	9.2	7.1	9.8	6.2	11.9	10.2	7.5	6.7
Level of significance								
Inter-individual variability for amyloid negative (A−) participants								
RSFvsNonRSF		^⁎⁎^		^⁎⁎^		^⁎⁎^		^⁎⁎^
BSvsCER					^⁎⁎^	^⁎⁎^		
WCvsCER							ns	ns
SUVrvsBP			^⁎^	^⁎⁎^				
Comparison within A− group								
RSFvsNonRSF		^⁎⁎^		^⁎⁎^		^⁎⁎^		ns
BSvsCER					^⁎⁎^	^⁎⁎^		
WCvsCER							^⁎⁎^	^⁎⁎^
SUVrvsBP			^⁎⁎^	^⁎⁎^				
Comparison within A+ group								
RSFvsNonRSF		^⁎⁎^		^⁎⁎^		^⁎⁎^		^⁎⁎^
BSvsCER					^⁎⁎^	^⁎⁎^		
WCvsCER							^⁎⁎^	^⁎⁎^
SUVrvsBP			^⁎⁎^	^⁎⁎^				

ns: nonsignificant; ^⁎^: *p* < 0.05; ^⁎⁎^: *p* < 0.0005, therefore remain significant after Bonferroni correction for multiple comparisons. The lower portion of the table reports the significance level for pair-wise comparison between different quantification techniques. For comparisons between RSF partial volume corrected methods with their counterparts without RSF PVC (RSFvsNonRSF), the significance level is reported under the RSF column; for comparisons between brainstem referencing methods with their counterparts with the default cerebellar gray referencing (BSvsCER), the significance level is reported under the BS column; for comparisons between whole cerebellum referencing methods with their counterparts with the default cerebellar gray matter referencing (WCvsCER), the significance level is reported under the WC column; For time window comparison (4070vs3060), the significance level is reported under the 4070 column; for comparison between SUVr and BP measurements (SUVrvsBP), the significance level is reported under the SUVr column. It should be noted that comparison for a particular technical variability was only performed between otherwise equivalent techniques.

### 3.3 Longitudinal knight ADRC cohort analysis

**Fig. 3:**
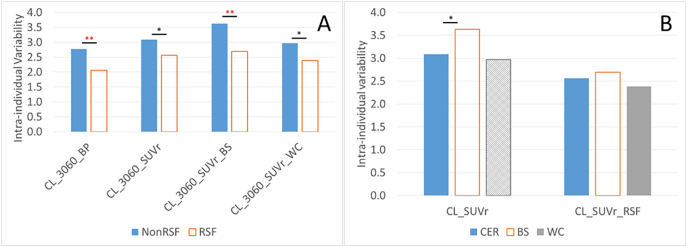
Comparison of the intra-individual variability of amyloid burden measurements for quantification methods with and without partial volume correction (A), and using different reference region (B). RSF: Regional spread function based partial volume correction; NonRSF: without partial volume correction; **significant difference (*p* < 0.0005) that survives Bonferroni correction for multiple comparisons. *significant difference (*p* < 0.05) that does not survive Bonferroni correction. The analysis was based on the longitudinal Knight ADRC data for stable amyloid negative participants.

The results of the analysis of the longitudinal Knight ADRC cohort are summarized in Table 4 and Fig. 3. Based on this analysis, application of RSF PVC, in general, lead to lower intra-individual variability in the amyloid-negative group (Fig. 3A) and larger effect size in longitudinal changes in the amyloid-positive group. The lowest variability in the amyloid-negative group (2.1 CL) was observed when binding potential analysis with RSF PVC (CL_3060_BP_RSF) was used to quantify amyloid burden with cerebellar gray matter referencing. The observed annual changes in amyloid burden had the largest effect size (1.9) when brainstem was used as the reference region and SUVr analysis was performed with RSF PVC (CL_3060_SUVr_RSF_BS). For this approach, its largest observed measured annual change (5.2 Centiloid units) resulted in the smallest sample size (*N* = 17 per arm) in a hypothetical anti-amyloid clinical trial that reduced the rate of amyloid accumulation by 50%, although the intra-individual variability was larger. All quantification methods detected longitudinal accumulation (*p* < 0.0005) of amyloid in the amyloid positive group.

**Table 4:** Summary of Centiloid analysis results in the longitudinal ADRC cohort.

	CL_3060_BP	CL_3060_ BP_RSF	CL_3060_ SUVr	CL_3060_ SUVr_RSF	CL_3060_ SUVr_BS	CL_3060_ SUVr_RSF _BS	CL_3060_ SUVr_WC	CL_3060_ SUVr_RSF _WC
Intra-individual variability	2.8	2.1	3.1	2.6	3.6	2.7	3.0	2.4
Annualized rate of change in A+ group	3.5	3.7	3.9	4.4	5.1	5.2	4.0	4.3
Effect size of annual change	1.3	1.8	1.2	1.7	1.4	1.9	1.3	1.8
Sample size (50% reduction in rate)	51	31	70	45	30	17	58	31
Level of significance								
Longitudinal change in A+ group	^⁎⁎^	^⁎⁎^	^⁎⁎^	^⁎⁎^	^⁎⁎^	^⁎⁎^	^⁎⁎^	^⁎⁎^
Test of intra-individual variability								
RSFvsNonRSF		^⁎⁎^		^⁎^		^⁎⁎^		^⁎^
BSvsCER					^⁎^	ns		
WCvsCER							ns	ns
SUVrvsBP			ns	^⁎^				

ns: nonsignificant; ^⁎^: *p* < 0.05; ^⁎⁎^: *p* < 0.0005, therefore remain significant after Bonferroni correction for multiple comparisons. For comparisons between RSF partial volume corrected methods with their counterparts without RSF PVC (RSFvsNonRSF), the significance level is reported under the RSF column; for comparisons between brainstem referencing methods with their counterparts with the default cerebellar gray referencing (BSvsCER), the significance level is reported under the BS column; for comparisons between whole cerebellum referencing methods with their counterparts with the default cerebellar gray matter referencing (WCvsCER), the significance level is reported under the WC column; For time window comparison (4070vs3060), the significance level is reported under the 4070 column; for comparison between SUVr and BP measurements (SUVrvsBP), the significance level is reported under the SUVr column. It should be noted that comparison for a particular technical variability was only performed between otherwise equivalent techniques.

### 3.4 Amyloid positivity thresholds

**Fig. 4:**
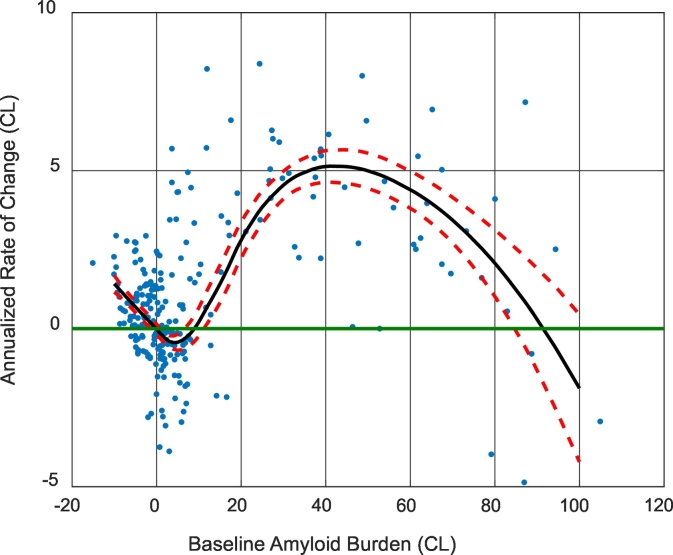
The annualized rate of change as a function of baseline amyloid burden. Dots are the raw data points, black solid lines are the average curve and red dash lines are 95% CI (based on bootstrapping). Note the minimum of the black curve (even the upper 95% CI curve) had a negative rate of change.

**Fig. 5:**
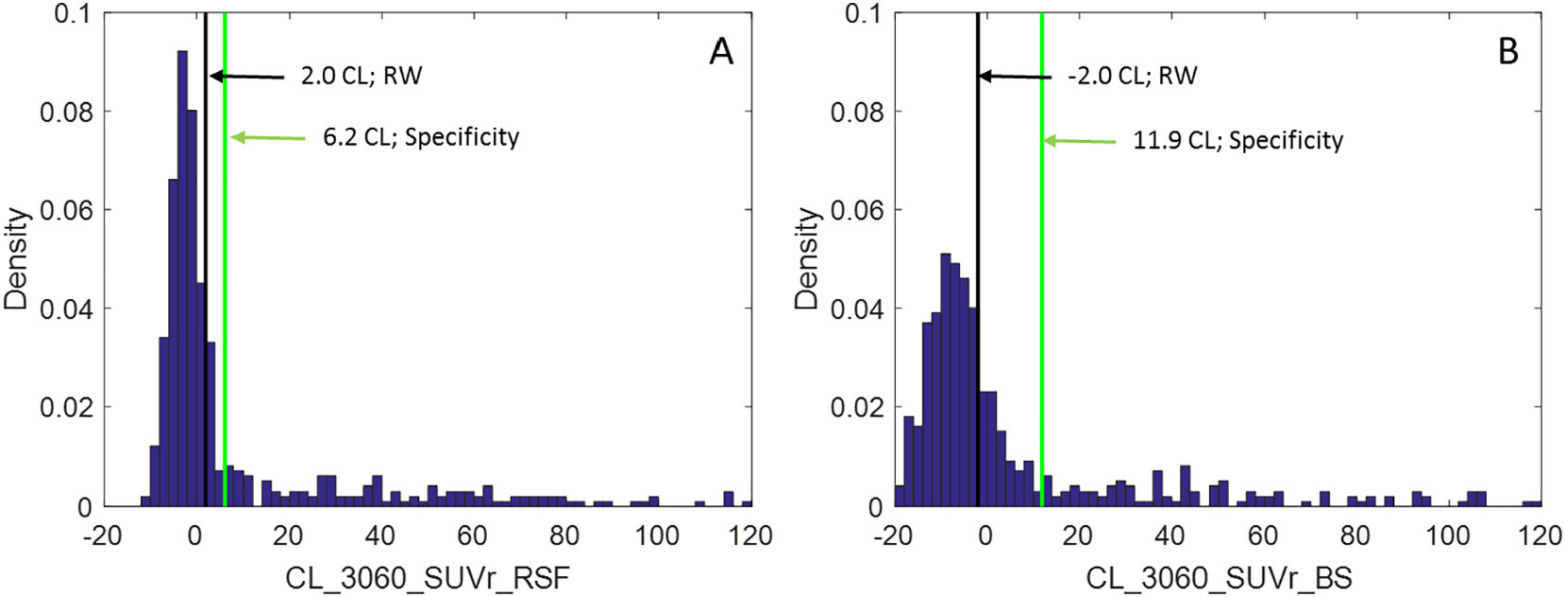
Histogram of the distribution of cognitively normal participants in the ADRC cohort as a function of amyloid burden and the amyloid positivity thresholds determined using the modified reliable worsening (RW) method (black) and the specificity method (green). The methods for PiB PET quantification are SUVr analysis with RSF partial volume correction using cerebellum cortex as the reference region and 30–60 min post-injection time window (CL_3060_SUVr_RSF) (A); and SUVr analysis without partial volume correction using brainstem as the reference region and 30–60 min post-injection time window (CL_3060_SUVr_BS) (B).

When the Centiloid conversion is applied to empirical amyloid positivity thresholds our group has previously used, a mean cortical BP_ND_ threshold of 0.18 is transformed into 18.2 CL, and a mean cortical SUVr_RSF threshold of 1.42 is transformed into 16.4 CL. The specificity approach based amyloid positivity thresholds are listed in Table 2, Table 3, and the thresholds were in the range of 6–12 Centiloid units. The procedure for the determination of RW threshold is illustrated in Fig. 4. The modified RW approach based thresholds are listed in Table 3 and varied from −2.0 to 11.0 CL. These RW thresholds were all integer values because we only sampled the rate versus baseline amyloid burden curves at integer CLs. Both the specificity and the RW based approaches generated thresholds that were more sensitive (i.e. lower in Centiloid units) than our empirical thresholds, which were more geared toward separation of clinical AD patients from the general population. The specificity and RW based thresholds in relation to the distribution of amyloid burden in the Knight ADRC cohort are displayed in Fig. 5 for two example analytic approaches. Using the lowest threshold in CL (−2.0 for CL_3060_SUVr_BS determined with the RW approach), 46% of the cognitively normal participants in their 70s were classified as amyloid positive; and with a threshold of 11.9 CL (determined using the specificity approach for CL_SUVr_BS), 36% of the cognitive normal 70-year-olds were classified as amyloid positive. In comparison, an 18.2 CL threshold derived from our empirical approach resulted in 33% of the cognitively normal 70-year-olds classified as amyloid positive.

## 4 Discussion

The Centiloid approach is proposed and designed to standardize PET based amyloid burden measurements and the original Centiloid paper suggested five benefits that widespread use of the Centiloid approach would bring: 1) facilitation of direct comparisons of results across different research groups when methods and tracers are involved; 2) a clear definition of amyloid positivity thresholds; 3) further definition of meaningful amyloid burden ranges; 4) an expression of longitudinal changes in standard units; 5) direct comparisons of the characteristics of different tracers (Klunk et al., 2015). As amyloid PET tracers are designed for in vivo measurement of the underlying amyloid pathology, studies have found a good correlation between PET measurement and post-mortem pathology (Clark et al., 2011; Ikonomovic et al., 2008). Therefore, the Centiloid approach allows intuitive interpretation of the amyloid burden measurements in the Centiloid scale. For example, a 20 CL would indicate the participant had approximately 20% amyloid plaque density of an average AD patient. To assess whether the proposed benefits of the Centiloid approach are achievable, we calibrated 13 versions of PiB PET analysis techniques following the Centiloid working group's recommendation and then examined the impact of variations in analytic approach to the signal to noise ratio and sensitivity of the estimated brain amyloid burden after they were converted into CL. Thresholds for amyloid positivity in CL were also determined using three different approaches.

As demonstrated in the original Centiloid paper (Klunk et al., 2015) and replicated in our study, the Centiloid approach successfully normalized the mean amyloid burden to approximately 0 for the YC group and approximately 100 for the AD group for the GAAIN dataset regardless which quantification method is used (Table 2, Fig. 1). When applied to the larger Knight ADRC cohort, the measured amyloid burden ranges from approximately 0 CL in amyloid negative participants to an average of about 60 CL in amyloid positive participants for all methods compared (Table 3). However, when tested statistically, differences can be detected in both mean amyloid burden and variability when different quantification methods are adopted even after conversion to the Centiloid scale. It was observed in the Knight ADRC data that the peak distribution of the Centiloid measure (Fig. 5) and the average for amyloid negative participants (Table 3) were below zero CL for many analysis methods. While this might be partly attributable to selection bias, as we first used a threshold to define amyloid positivity that biased the average amyloid burden measurement toward the lower end, the primary reason for this phenomenon was likely due to the differences in nonspecific binding of the tracer and tracer delivery differences between younger controls and older controls. These observations suggest that, with the current approach to generate amyloid burden measurements in the Centiloid scale, the proposed benefits 1 and 4 can be achieved on a qualitative level while statistically detectable differences exist and need to be addressed. As an example, if we want to compare the amyloid burden of a sporadic AD population (Knight ADRC) to the amyloid burden of autosomal dominant AD population in the DIAN cohort, statistical difference due to the difference in imaging protocol can be expected, i.e. 30–60 min post-injection window in Knight ADRC vs. 40–70 min post-injection window in DIAN, even after conversion into the Centiloid scale. It should be noted that this difference may be caused in part by the relatively small sample size of the calibration dataset to generate the Centiloid conversion equations.

As a biomarker for amyloid pathology, the imaging based continuous measure of amyloid burden was often dichotomized into normal or abnormal based on a threshold for clinical diagnosis/staging of AD (Jack et al., 2011; Sperling et al., 2011), as eligibility criteria for clinical trials (Hampel et al., 2015; Sperling et al., 2014), and to facilitate research (Jack et al., 2016; Vlassenko et al., 2016). In this study, we determined amyloid positivity thresholds in CL using three different approaches. Although, there was substantial variability in the numerical values of Centiloid thresholds for amyloid positivity, the percentage of cognitively normal participants in their 70s classified as amyloid positive only range moderately from 33% to 46%. We did not observe a very high (and probably unrealistic) amyloid positivity percentage of 75% when the specificity approach was used to define the threshold as Jack et al. (Jack et al., 2017) reported. In general, the specificity-based thresholds were typically higher than the RW based thresholds (Table 3, Fig. 5) contrary to what Jack et al. reported with a specificity threshold of 8 CL and an RW threshold of 19 CL (Jack et al., 2017). These discrepancies might be a consequence of the analysis methods that were chosen to quantify amyloid as well as differences in the study cohorts and highlight the caution necessary for adopting a threshold obtained from a different processing method. This study demonstrates that the thresholds for amyloid positivity in CL were implementation dependent, especially when the RW method was used to determine the threshold. For example, when brainstem was used as the reference, the amyloid positivity threshold was −2.0 CL, and if we use this threshold on the GAAIN dataset quantified using the standard Centiloid processing, 20 of the 34 young controls would be classified as amyloid positive which was apparently incorrect. Therefore, the underlying implementation of the quantification approach has to be taken into account when interpreting the threshold, and one should not simply adopt a threshold that was developed under method A and applying it to quantification results obtained under method B even after conversion into CL. These observations suggest there are challenges in using the Centiloid approach to achieve the benefits 2 and 3 summarized at the beginning of our discussion and proposed in the original Centiloid paper (Klunk et al., 2015). However, it should be pointed out that part of the reason for the method dependent amyloid positivity thresholds was that the methodology for deriving the thresholds was driven more by the signal to noise ratio property of the amyloid burden measurements rather than the underlying amount of pathology. The current analysis suggests that the signal to noise ratio property is very much method dependent, and as a simple linear transformation, one should not expect the Centiloid conversion procedure to remove the differences in signal to noise ratio property inherent in the quantification methods. Although this should be obvious for people familiar with imaging quantification and the implementation of the Centiloid approach, it may not be as evident for many people in the research community using amyloid PET as a tool. On the other hand, if the goal is to define physiologically meaningful amyloid positivity thresholds or ranges of amyloid burdens, then one should use common Centiloid values for the definition of the thresholds and ranges.

For longitudinal studies, the different implementation of amyloid PET imaging quantification approaches led to different sensitivity to longitudinal changes even after applying the Centiloid conversion. This difference in sensitivity resulted in different sample sizes needed for hypothetical clinical trials aiming at slowing amyloid accumulation. We acknowledge the fact that some of the stable amyloid negative participants, based on whom the intra-individual variability in amyloid burden measurement was assessed, may express real albeit small accumulation of amyloid and therefore our intra-individual variability is slightly biased toward a small positive change over time; however it allowed us to provide a reasonable approximation of the noise in the amyloid burden measurements in longitudinal studies as we used a very conservative amyloid burden threshold and only included people who remained amyloid negative at both visits.

The last benefit of adopting the Centiloid approach was allowing for a direct comparison of the characteristics of different tracers (Klunk et al., 2015), as the Centiloid approach converts all measurements into the same scale. We believe this can be applied to the comparison of different quantification as well, and as we observed in this study, different quantification methods have different sensitivity and specificity properties. After converting to the common Centiloid scale, it is easier to draw intuitive conclusions about the benefits and drawbacks of the different approaches. For example, the RSF PVC technique consistently reduced variability in the measurements and improved sensitivity to longitudinal changes.

## 5 Conclusion

Using the Centiloid approach to convert quantitative amyloid burden measurements into the Centiloid scale brings the amyloid measurements into a comparable dynamic range. Meanwhile, the Centiloid value derived from different analysis techniques inherits most of the inherent characteristics of the underlying analytic approaches, and these differences are detectable in the analysis of large datasets and lead to different sensitivity to amyloid burden changes in longitudinal studies. Because of these differences, the amyloid positivity thresholds derived from different analysis techniques differ from one technique to another. Therefore, when amyloid measurements obtained from different centers are combined for analysis, the impact of the differences in underlying acquisition protocols and analysis techniques need to be taken into consideration even after conversion into Centiloid scale.

## Appendix A Supplementary data

Image 1

SUVR and Centiloid values for the GAAIN dataset. Image 2
