# Supplementary material for: Utilizing the Centiloid scale in cross-sectional and longitudinal PiB PET studies
Source: Neuroimage Clin. 2018 Apr 25;19:406–16. doi: 10.1016/j.nicl.2018.04.022 (PMC6051499; doi:10.1016/j.nicl.2018.04.022)
Supplement: Supplementary file 1 [file mmc1.docx]

**Supplementary material.**

**Centiloid Analysis for the GAAIN Dataset**

The Centiloid measures derived from our local implementation of the standard Centiloid analysis procedure strongly agree with the published Centiloid values for the GAAIN dataset (Supp. Fig. 1; Supp. Table 1).


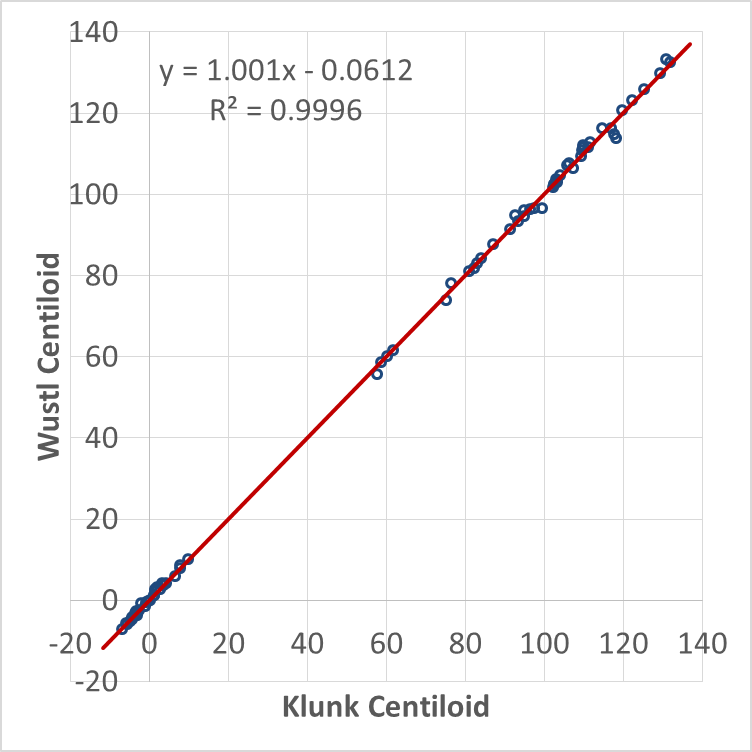


Supplementary Figure 1. Comparison between amyloid burden in the Centiloid scale derived from our local implementation of the standard Centiloid analysis and the reported Centiloid values in (Klunk et al., 2015) for the GAAIN dataset.

The conversion equation from our local Centiloid SUVR to the Centiloid scale is:

$CL\_WUSTL=100\times(SUVR_{CL}-1.016)/1.071$ supp. eq. 1

Global indices of amyloid burden obtained from the twelve different analysis techniques was listed in Supp. Table 2. Following level-2 procedure described in Klunk et al., the equation for conversions from individual amyloid burden measurements to Centiloid scale were:

$CL\_3060\_BP=126.7\times PiB\_3060\_BP-4.4$ supp. eq. 2$CL\_3060\_BP\_RSF=53.9\times PiB\_3060\_BP\_RSF-4.7$ supp. eq. 3$CL\_4070\_BP=122.5\times PiB\_4070\_BP-6.2$ supp. eq. 4$CL\_4070\_BP\_RSF=52.0\times PiB\_4070\_BP\_RSF-4.2$ supp. eq. 5$CL\_3060\_SUVR=111.8\times PiB\_3060\_SUVR-119.3$ supp. eq. 6$CL\_3060\_SUVR\_RSF=45.0\times PiB\_3060\_SUVR\_RSF-47.5$ supp. eq. 7$CL\_4070\_SUVR=102.8\times PiB\_4070\_SUVR-112.2$ supp. eq. 8$CL\_4070\_SUVR\_RSF=40.7\times PiB\_4070\_SUVR\_RSF-42.9$ supp. eq. 9$CL\_3060\_SUVR\_BS=172.5\times PiB\_3060\_SUVR\_BS-120.0$ supp. eq. 10$CL\_3060\_SUVR\_RSF\_BS=84.8\times PiB\_3060\_SUVR\_RSF\_BS-47.9$ supp. eq. 11$CL\_3060\_SUVR\_WC=122.9\times PiB\_3060\_SUVR\_WC-119.1$ supp. eq. 12$CL\_3060\_SUVR\_RSF\_WC=53.6\times PiB\_3060\_SUVR\_RSF\_WC-46.2$ supp. eq. 13

After conversion to Centiloid scale using the above equations, Centiloid values were also reported in Supp. Table 2.

**References**

Klunk, W.E., Koeppe, R.A., Price, J.C., Benzinger, T.L., Devous, M.D., Sr., Jagust, W.J., Johnson, K.A., Mathis, C.A., Minhas, D., Pontecorvo, M.J., Rowe, C.C., Skovronsky, D.M., Mintun, M.A., 2015. The Centiloid Project: standardizing quantitative amyloid plaque estimation by PET. Alzheimers Dement 11, 1-15.e11-14.
